# Hypoxemic Respiratory Failure from Acute Respiratory Distress Syndrome Secondary to Leptospirosis

**DOI:** 10.1155/2017/9062107

**Published:** 2017-10-12

**Authors:** Shannon M. Fernando, Pierre Cardinal, Peter G. Brindley

**Affiliations:** ^1^Division of Critical Care, Department of Medicine, University of Ottawa, Ottawa, ON, Canada; ^2^Department of Emergency Medicine, University of Ottawa, Ottawa, ON, Canada; ^3^Department of Critical Care Medicine, University of Alberta, Edmonton, AB, Canada

## Abstract

Acute respiratory distress syndrome (ARDS), characterized by hypoxemic respiratory failure, is associated with a mortality of 30–50% and is precipitated by both direct and indirect pulmonary insults. Treatment is largely supportive, consisting of lung protective ventilation and thereby necessitating Intensive Care Unit (ICU) admission. The most common precipitant is community-acquired bacterial pneumonia, but other putative pathogens include viruses and fungi. On rare occasions, ARDS can be secondary to tropical disease. Accordingly, a history should include travel to endemic regions. Leptospirosis is a zoonotic disease most common in the tropics and typically associated with mild pulmonary complications. We describe a case of a 25-year-old male with undiagnosed leptospirosis, presenting with fever and severe hypoxemic respiratory failure, returning from a Costa Rican holiday. There was no other organ failure. He was intubated and received lung protective ventilation. His condition improved after ampicillin and penicillin G were added empirically. This case illustrates the rare complication of ARDS from leptospirosis, the importance of taking a travel history, and the need for empiric therapy because of diagnostic delay.

## 1. Introduction

Acute respiratory distress syndrome (ARDS) is an intense inflammatory lung condition that is triggered by a wide range of pulmonary or systemic insults to the alveolar-capillary membrane [1]. This results in increased vascular permeability, which in turn causes alveolar and interstitial edema and hypoxemia. Prevalence of ARDS in the Intensive Care Unit (ICU) has been estimated as high as 10%, with in-hospital mortality ranging from 30 to 50%, depending on the severity of illness [2]. ARDS is defined by the Berlin Criteria [3], which incorporates four components: (a) acute onset; (b) hypoxemia (defined as a PaO_2_/FiO_2_ ratio of <300); (c) bilateral infiltrates on chest radiograph; and (d) absence of cardiac failure. While the most common causes include direct lung injury (pneumonia or aspiration), extrapulmonary sepsis, and trauma [2], the etiologic differential for ARDS is large. This means that the clinician should be prepared to undertake a thorough history and remain open to atypical causes.

There are multiple infectious triggers for ARDS. These include the aforementioned pulmonary infections (bacterial, viral, or fungal), and the disseminated inflammatory response associated with sepsis [4]. Various tropical diseases can also cause ARDS, and in such cases, the most commonly implicated etiology is the parasite falciparum malariae [5]. While even more rare, ARDS has also been associated with the tropical disease leptospirosis. This disease is typically associated with nonspecific constitutional symptoms but has the potential to precipitate multisystem organ failure and death [6]. Only a handful of published reports of ARDS secondary to leptospirosis exist [7–9]. We present a case of a patient eventually diagnosed with leptospirosis, but who presented with isolated hypoxemic respiratory failure, and who fulfilled criteria for ARDS. This patient did not get better until he received a full travel history, which in turn led to empiric ampicillin and penicillin G. This case highlights the importance of taking a travel history and considering the rare but potentially deadly diagnosis of leptospirosis.

## 2. Case Report

**Figure 1:**
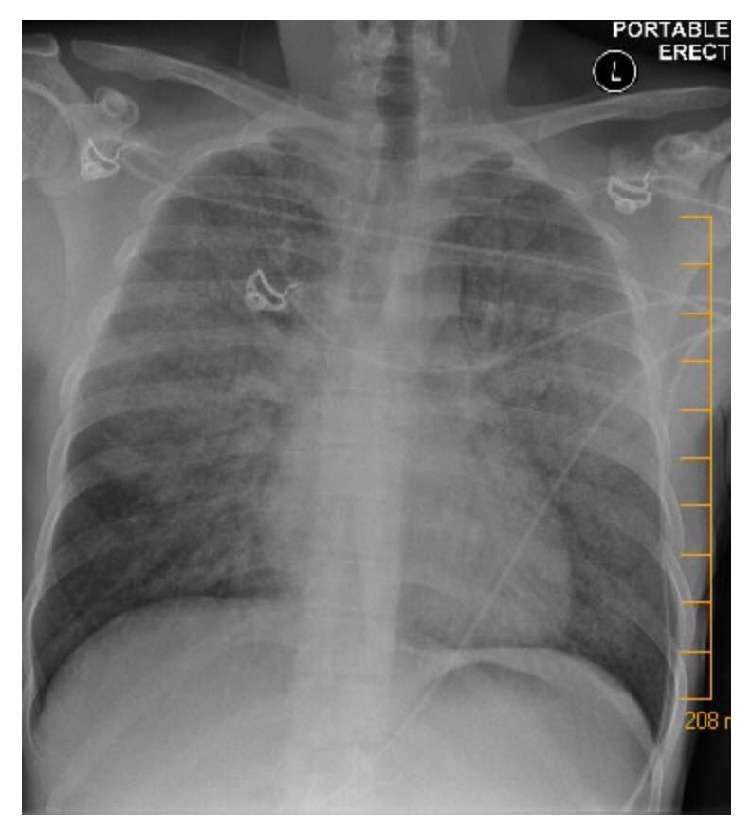
Anterior-posterior portable chest radiograph demonstrating bilateral pulmonary opacities, consistent with ARDS.

A 25-year-old male with no previous medical comorbidities was brought to the Emergency Department (ED) with increasing tachypnea and dyspnea over the previous day. The accompanying partner reported that the patient had been intermittently warm to the touch for the past week, complaining of myalgia and headache. He has never used any medications. The pair had just returned to Canada from two weeks in Costa Rica, where the patient had begun feeling unwell one day before flying home. Immediately upon returning to Canada, the patient saw his primary care physician, who believed he had influenza. In the following days, the patient's condition worsened and he presented to the ED with the following vital signs: blood pressure of 125/75 mmHg, heart rate (HR) of 110 beats/min, respiratory rate (RR) of 35 breaths/minute, temperature of 38.6 degrees Celsius, and oxygen saturation of 82%. Glasgow Coma Scale (GCS) was 15/15. He was in obvious respiratory distress with accessory muscle use. A portable chest radiograph demonstrated bilateral opacities (Figure 1). The patient was placed on a nonrebreather (FiO_2_ of 1), but his work of breathing did not substantially improve, and he remained hypoxemic. Therefore, the patient was intubated in the ED and an arterial line was inserted. ED bloodwork revealed a pO_2_ of 65 mmHg on 100% oxygen, but normal electrolytes, renal function, liver enzymes, and coagulation parameters. Following ICU transfer, the patient was diagnosed with ARDS due to his severely depressed PaO_2_/FiO_2_ ratio (110 upon ICU admission), bilateral opacities, and no signs that would suggest cardiac dysfunction (i.e., normal blood pressure, no peripheral edema, and normal electrocardiogram). He was ventilated with a standardized ARDSNet protocol that focused on low tidal volume (5 mL/kg of ideal body weight and a target pH > 7.25), plus a positive end-expiratory pressure (PEEP) increased to 10 mmHg [10, 11]. While the etiology for ARDS was unclear, it was presumed infectious in origin, given his fevers and travel. Blood and urine cultures were performed, along with bronchoalveolar lavage (BAL). Bronchoscopy did not show any pulmonary hemorrhage or purulent secretions. Computed tomography (CT) of the chest confirmed bilateral edema but did not reveal any lobar infiltrate consistent with bacterial pneumonia. Transthoracic echocardiogram revealed normal biventricular function, with no vegetation or valvular regurgitation.

Infectious Diseases consultation led to initiation of broad-spectrum antimicrobials (piperacillin-tazobactam, azithromycin, and vancomycin). Initial blood, urine, and BAL cultures grew no pathogens. Malaria thick and thin smears were performed several times and were negative. Dengue serology was also negative, as was human immunodeficiency virus (HIV) and hepatitis A, B, and C. Despite broad antimicrobial treatment, the patient's respiratory status worsened, which led to prone positioning [12].

Given the recent travel history to Costa Rica, the diagnosis of leptospirosis was considered, but after a delay of 48 hours from presentation. The clue was that, following further questioning, the patient's partner reported that she and the patient had spent several days swimming in freshwater, which they had also drunk. Both are sources of leptospirosis transmission [6, 13]. Leptospirosis serology was sent (specifically, an IgM-detection enzyme-linked immunosorbent assay). Given the prolonged time required to establish leptospirosis diagnosis via serology, the patient was empirically treated with ampicillin and penicillin G [14].

Over the next 48 hours, the patient's condition substantially improved. He became afebrile, his hypoxemia resolved, and his chest radiograph improved. Otherwise, his blood work remained largely unchanged, with normal renal, hepatic, and coagulation function. He was extubated on postadmission day five, was transferred to the medicine ward on day six, and was discharged home on day eight. Diagnosis was confirmed but not until after discharge: due to a positive IgM for* Leptospira* and a* Leptospira canicola* titer of 1 : 200. Diagnosis was further confirmed with microscopic agglutination testing (MAT). He was seen in follow-up by the Infectious Diseases service and never demonstrated any renal dysfunction, hepatic dysfunction, or coagulopathy suggesting Weil's disease.

## 3. Discussion

Leptospirosis is a zoonotic disease caused by* Leptospira*. These are highly mobile, obligate aerobic spirochetes with features in common with both gram-positive and gram-negative bacteria [6]. This spirochete has been found worldwide, but most commonly in the tropics [13]. It is commonly transmitted from contaminated freshwater in endemic regions, or from animals, such as rodents and bats. The range of disease manifestation is vast and includes those with positive serology but no symptoms or those with minimal symptoms living in endemic regions [15].

Leptospirosis is most often characterized by a nonspecific febrile illness, and therefore it can be difficult to distinguish [16]. The disease can be associated with high mortality, but it is difficult to establish accurate morbidity and mortality rates [13]. Severe leptospirosis can result in Weil's disease: characterized by jaundice, renal dysfunction, and coagulopathy [17]. This can progress to multisystem organ failure, rhabdomyolysis, and death. Definitive diagnosis of leptospirosis requires recovery of leptospires either by culture or by immunohistochemical staining. Serology can also be performed using MAT or IgM-detection. Regardless of the method used, results can take days to weeks, and, therefore, if there is a high degree of suspicion, patients should receive empiric treatment. There is a paucity of evidence regarding optimal antimicrobials for leptospirosis, but sources recommend doxycycline for prophylaxis/mild disease [18, 19] and ampicillin and penicillin G for severe disease [14]. Ceftriaxone is a suitable alternative for treatment of severe disease and in patients with a penicillin allergy [20].

Pulmonary manifestations of leptospirosis have been reported, but are typically mild, and occur in conjunction with other failing organs [8, 21]. Severe ARDS has been described in a few cases reports of leptospirosis [7, 8], though again this is in the context of sepsis and multisystem organ failure. Other cases describe ARDS occurring secondary to pulmonary hemorrhage in leptospirosis [22, 23]. Of note, our patient demonstrated no evidence of hemorrhage on clinical history, CT scan, or bronchoscopy.

The mechanism by which leptospirosis triggered ARDS (in the absence of sepsis) is unclear, though two theories have been proposed to explain the intense inflammatory response [24]. The first is a toxin-mediated capillary vasculitis [25]. This is supported by pathologic evidence that, in patients that die with pulmonary complications, lung tissue has significantly less leptospires than liver and blood counts. In other words, the belief is that pulmonary abnormalities may occur secondary to circulating toxins produced by the pathogen at distant sites [13]. The second proposed mechanism is immune-mediated. Prominent inflammatory mediators, such as cytokines, have been demonstrated to be elevated in leptospirosis [26]. Following this mechanism, postmortem alveolar light microscopy shows a predominance of macrophages, lymphocytes, and plasma cells [27].

Our patient was diagnosed with ARDS but assumed to have a typical bacterial etiology. Leptospirosis was not even contemplated until 48 hours after admission. This is because of its very rare prevalence in North America, coupled with his nonspecific symptoms. Initial diagnostic work-up should include blood cultures, urine cultures, sputum cultures (if available), serological testing (for intracellular bacteria), and microbial sampling of the lung, ideally by BAL [4]. Advanced imaging, such as CT of the chest, may also be considered if the patient is safe for transport.

Community-acquired bacterial pneumonia remains the leading cause of ARDS [1], and therefore antimicrobial coverage of both typical and atypical organisms is prudent. Of note, leptospirosis should be susceptible to piperacillin-tazobactam, though it did not appear to ameliorate our patient. However, it is believed that there is variability in susceptibility to various antimicrobials, based on geographic variation [28]. Special attention should be paid to immunocompromised individuals, as they have greater propensity for fungal or parasitic organisms. Other history should include sick contacts, pets, exposures (i.e., farming, occupational), and particularly travel. Our patient's recent travel to an endemic leptospirosis region led to empiric therapy.

There is no evidence that ventilatory management of patients with ARDS from leptospirosis should be any different from other ARDS patients. These patients benefit from a lung protective strategy, marked by low tidal volumes and with the option for increasing PEEP [10, 11]. Fluid therapy should be sufficient to avoid cardiovascular collapse, but should be used with enough caution to avoid worsening pulmonary edema [29]. There is also some evidence for mechanical ventilation in the prone position [12]. Given the patient's age, lack of medical comorbidities, and single-system failure, he may have been considered for extracorporeal membrane oxygenation (ECMO), should his condition have worsened [30].

## 4. Conclusion

ARDS is a life-threatening condition. In addition to providing mechanical ventilation and fluid therapy that is minimally injurious, clinicians should also identify and target the etiology driving the inflammatory process. A few case reports have identified leptospirosis as the trigger for ARDS. However, given the nonspecific symptoms, diagnosis in North America is only likely to follow a thorough travel history. Furthermore, treatment will need to be empiric, as definitive laboratory diagnosis will be delayed days to weeks. We describe a case of ARDS in a patient with undiagnosed leptospirosis and no other end-organ dysfunction. Making note of the patient's recent travel history, and his exposure to tropical freshwater, heightened suspicion enough that he received timely empiric treatment and made a full recovery.
